# Quantifying microstructural dynamics and electrochemical activity of graphite and silicon-graphite lithium ion battery anodes

**DOI:** 10.1038/ncomms12909

**Published:** 2016-09-27

**Authors:** Patrick Pietsch, Daniel Westhoff, Julian Feinauer, Jens Eller, Federica Marone, Marco Stampanoni, Volker Schmidt, Vanessa Wood

**Affiliations:** 1 Laboratory of Nanoelectronics, Department of Information Technology and Electrical Engineering, Gloriastrasse 35, ETH Zurich, 8092 Zurich, Switzerland; 2 Institute of Stochastics, Helmholtzstrasse 18, Ulm University, 89069 Ulm, Germany; 3 Swiss Light Source, WBBA/216, Paul Scherrer Institut, 5232 Villigen, Switzerland; 4 Institute for Biomedical Engineering, Department of Information Technology and Electrical Engineering, Gloriastrasse 35, University and ETH Zurich, 8092 Zurich, Switzerland

## Abstract

Despite numerous studies presenting advances in tomographic imaging and analysis of lithium ion batteries, graphite-based anodes have received little attention. Weak X-ray attenuation of graphite and, as a result, poor contrast between graphite and the other carbon-based components in an electrode pore space renders data analysis challenging. Here we demonstrate *operando* tomography of weakly attenuating electrodes during electrochemical (de)lithiation. We use propagation-based phase contrast tomography to facilitate the differentiation between weakly attenuating materials and apply digital volume correlation to capture the dynamics of the electrodes during operation. After validating that we can quantify the local electrochemical activity and microstructural changes throughout graphite electrodes, we apply our technique to graphite-silicon composite electrodes. We show that microstructural changes that occur during (de)lithiation of a pure graphite electrode are of the same order of magnitude as spatial inhomogeneities within it, while strain in composite electrodes is locally pronounced and introduces significant microstructural changes.

## Abstract (web summary)

Tomographic imaging of graphite-based anodes is challenging due to weak X-ray attenuation contrast. Here, the authors use operando propagation-based phase contrast tomography and digital volume correlation to study the electrochemical activity and microstructural dynamics in (silicon−) graphite electrodes.

## Graphical abstract

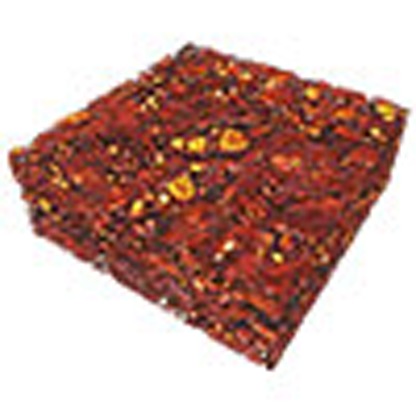

During lithium ion battery (LIB) operation, lithium ions react with the electrochemically active material in the porous electrodes, driving morphological changes in the active materials, which in turn can influence the electrode microstructure^1^. Not only electrochemical performance is dependent on material morphology and electrode microstructure, but electrochemical degradation and failure is often associated with non-reversible changes to the microstructure or inhomogeneities within it (refs 2, 3). To improve LIB performance and longevity, it is therefore crucial to understand local electrochemical activity and the resulting changes in lithium distribution within the electrodes, as well as dynamic morphological changes occurring in the electrode microstructures during battery operation. This manuscript presents a generalized method by which the electrochemical activity and microstructural dynamics during battery operation can be tracked. We examine graphite-containing electrodes because they are the most prevalent type of LIB anodes and present several challenges to visualization and quantification that until now have not been overcome.

Synchrotron-based X-ray tomographic microscopy (XTM) has received widespread attention in the field of LIBs as it allows for non-invasive imaging of electrode microstructures with high spatial and temporal resolution. This technique has been used for *ex-situ* microstructure and morphological studies of pristine and cycled electrodes^3^,^4^,^5^,^6^,^7^,^8^,^9^,^10^, as well as to track battery materials and electrodes during electrochemical cycling *in-situ* or *in-operando*^2^,^11^,^12^,^13^,^14^,^15^.

Because its low core charge number (Z=3) makes lithium highly transparent to X-rays, XTM experiments that map the lithium content in battery materials during (de)lithiation typically track: (i) the volumetric change, (ii) the attenuation coefficient change or (iii) the absorption edge shifts of the active material. Approaches (i) and (ii) can be performed with X-rays at a single energy and rely on the change in the number density of active material atoms (volume is inversely proportional to density and the X-ray attenuation coefficient is proportional to density). Approach (iii) is based on absorption spectroscopy and relies on the change of oxidation state of one or more species of host atoms. Analysing the lithium distribution in an electrode with any of these three techniques requires that the active material be distinguishable from other solid phases and from the pore space within the electrodes.

For these reasons, *in-situ* XTM studies have been predominantly carried out on electrode materials containing rather heavy elements. Techniques (i) and (ii) have been proven suitable for tin^2^,^13^ and germanium^12^ alloying anodes that undergo large volumetric and attenuation coefficient changes beyond 200% (refs 2, 12, 16) on lithiation, which allows the lithium distribution to be deduced rather precisely. Technique (iii) has been most successfully applied to track the oxidation state changes of transition metals in cathode materials on lithiation^17^.

For graphite, none of these three techniques is promising. First, carbon (Z=6) is weakly attenuating for hard X-rays, resulting in low contrast between it and the electrolyte-filled pore space. This in combination with edge enhancement artefacts in such systems renders data binarization particularly challenging. Secondly, even if binarization is possible, the volumetric and attenuation coefficient changes are only 10.5% (refs 1, 18, 19) and −11.4% (at 10 keV)^20^,^21^ respectively on full lithiation of graphite from C_6_ (delithiated) to LiC_6_ (lithiated), making intercalation hard to track and prone to segmentation errors. Finally, graphite does not exhibit any absorption edges in a feasible energy range^22^. It is therefore not surprising that optical studies on the lithiation dynamics of graphite have been the most successful ones so far, since the various Li_x_C_6_ phases change colour from black to blue to red to golden on lithiation^23^,^24^,^25^.

To investigate dynamic lithium distribution and microstructure evolution, we propose a twofold approach. First, we apply digital volume correlation (DVC)—previously used to study two-dimensional (2D) strains in graphite electrodes using an optical cell^25^, deformations in a Mn_2_O_4_ containing commercial coin cell^26^ and commercial primary batteries^27^- to the time-resolved set of tomographic data to precisely track even small deformations within the graphite electrodes as function of three-dimensional (3D) space and time. On the basis of these morphological changes, the electrochemical activity may be mapped throughout the electrodes. While a major advantage of DVC when applied to weakly attenuating materials is that it does not rely on error-prone data binarization procedures, binarization remains necessary for microstructural analysis.

Second, we therefore enrich the image contrast obtained with standard attenuation-based XTM measurements by making use of emerging X-ray phase contrast techniques. Coherent X-rays produced by synchrotrons probe the complex refractive index *n*=(1−*δ*)+*iβ* of a sample. While attenuation contrast tomography maps the imaginary part of the refractive index (*β*, representing beam attenuation), phase contrast tomography maps the real part of the refractive index (*δ*, representing beam deflection). Phase contrast tomography often requires more complicated measurement setups and is typically more prone to different types of artefacts than attenuation contrast tomography. However, it enhances the contrast between different weakly attenuating materials.

Different fully quantitative and semi-quantitative methods for phase contrast tomography have been developed^28^,^29^,^30^. Propagation-based phase contrast tomography using the phase retrieval algorithm by Paganin *et al*.^29^,^31^,^32^ is one of the simplest available techniques. Although paganin phase contrast (PPC) is fully quantitative only if (i) the sample consists of a single homogeneous material phase and a void phase and (ii) the ratio *δ*/*β* of the real and the imaginary parts of the material's X-ray refractive index is known, it has been applied to multiphase samples, including weakly attenuating lithium dendrites^5^, graphite electrodes^4^ and lithium sulphur electrodes^9^. In this case, *δ*/*β* can be considered a tuneable imaging parameter that determines the degree to which the phase is retrieved.

Here we present *operando* tomography measurements of graphite electrodes during (de)lithiation. Using PPC and optimizing the ratio *δ*/*β* only slightly compromises the effective resolution while allowing for improved contrast needed for binarization. DVC enables us to quantify the electrochemical activity throughout the electrodes, and, in combination with PPC, to perform a dynamic microstructure analysis. After validating this approach using well-studied graphite electrodes, we show that it can be applied to study the lithiation dynamics and structural evolution of graphite-silicon composite anodes for the next generation of LIBs.

## Results

### Paganin phase contrast tomography

**Figure 1:**
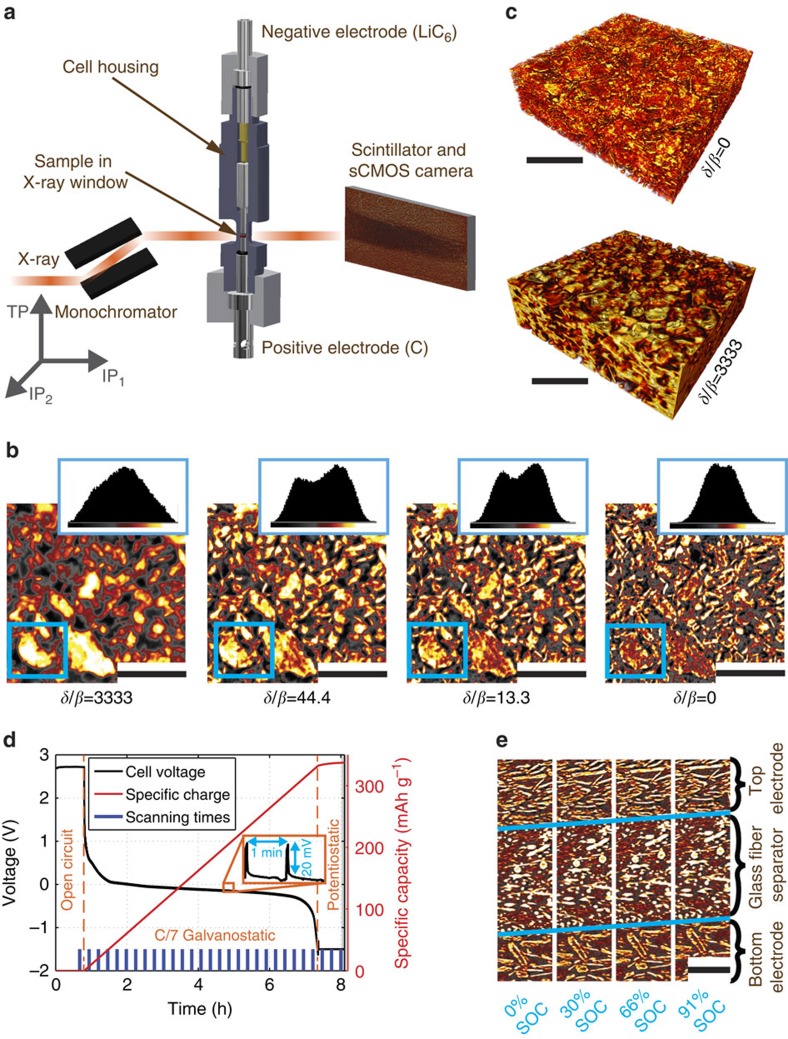
Measurement setup and parameter selection. (**a**) Sketch of the electrochemical cell and the measurement setup, including a coordinate system indicating the two IP directions and the TP direction. (**b**) Comparison of a slice in the bottom electrode, where the phase has been retrieved with different *δ*/*β* ratios. Insets show the respective colour-scale histograms. Scale bar length: 100 μm. (**c**) Microstructure rendering of a part of the bottom electrode using the retrieval parameters from the two extreme cases in (**b**). Scale bar length: 100 μm. (**d**) Specific charge, voltage and scanning times during *operando* electrochemical operation. The inset shows a zoom-in of the voltage profile arising from the applied galvanostatic intermittent titration technique protocol. (**e**) Vertical cuts through the sample at different states of charge. The blue lines mark the electrode–separator interfaces that shift on electrochemical operation. For better visibility, only a window around the separator is shown. Scale bar length: 50 μm.

Figure 1a schematically depicts the measurement setup. We construct an electrochemical cell for *operando* XTM, consisting of a polyether ether ketone cell housing tube and two steel current collectors. The system we investigate here consists of two, 250 μm-thick electrodes of platelet-shaped graphite particles spaced with a 250 μm-thick glass fibre separator. To capture both lithiation and delithiation dynamics simultaneously, the top electrode is fully lithiated before the experiment in a standard coin cell in half-cell configuration using a metallic lithium counter electrode. The incident 25 keV monochromatic X-rays penetrate the cell in transmission geometry and are converted to visible light using a scintillator such that the image can be magnified with an optical microscope and recorded by a sCMOS camera.

Figure 1b shows a slice through the pristine bottom electrode reconstructed using the Paganin algorithm with different coefficient ratios *δ*/*β* and the respective grey value histograms. The applied *δ*/*β* ratios provide a trade-off: higher ratios improve the grey value contrast between particles and background but also decrease the effective spatial resolution, which results in a loss of small features. 3D renderings of the bottom electrode for the two extreme cases *δ*/*β*=0 (pure attenuation contrast) and *δ*/*β*=3333 (strong PPC) in Fig. 1c confirm this observed trade-off. For this paper, we choose *δ*/*β*=13.3 to achieve reliable data binarization and preserve the microstructure details.

### *Operando* experiment

During the *operando* experiment, 29 tomographic, 15 min scans are continuously recorded (Fig. 1d), while the lithium ions are electrochemically moved from the top to the bottom electrode at an effective C/7 rate until the cut-off potential at −1.5 V is reached. We use a galvanostatic intermittent titration technique protocol^33^, where 57 s long current pulses alternate with 3 s long open circuit relaxation times. A potentiostatic step at the cut-off potential is added to ensure that all lithium ions migrate between the electrodes (Fig. 1d). Figure 1e shows corresponding subsections of vertical cuts through the tomograms at different states of charge (SOC). The SOC is computed by dividing the measured charge by the active mass of graphite multiplied with the theoretical capacity of LiC_6_ (372 mAh/g refs 34 and 35). On lithiation, the bottom electrode has slightly expanded in the through-plane (TP) direction, while the top electrode has contracted in the same direction on delithiation. This is in agreement with our understanding from literature that graphite reversibly expands by approximately 10.5% on full lithium intercalation^1^,^18^,^19^.

### Understanding electrode dynamics with DVC

To analyse the dynamics of the electrode, we apply DVC directly to the reconstructed data before any post-processing steps. DVC calculates a vector displacement field that maps two volumetric images locally on one another by maximizing the cross correlations between iteratively refined sub-volumes in the respective data sets. From the calculated vector fields, spatially resolved quantities like strain or relative volume expansion can be deduced.

**Figure 2:**
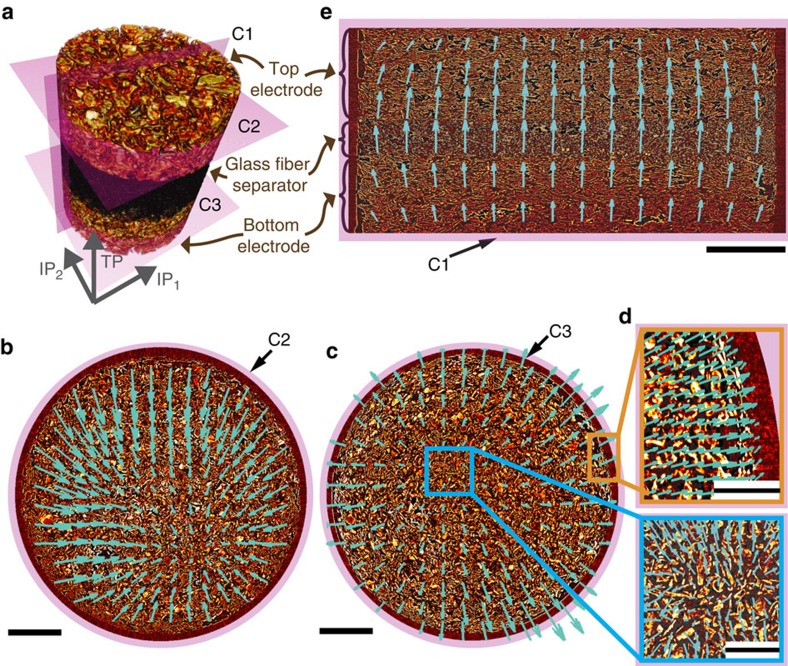
Digital volume correlation. (**a**) Schematic of the two electrodes, the separator (black) and the cuts C1, C2, and C3 (purple). The coordinate system indicates the two IP directions and the TP direction. (**b**) Top electrode with superimposed vector field indicating the IP electrode contraction from the first to the last time step (vectors are scaled by a factor 60). Scale bar length, 300 μm. (**c**) Analogue graphic for the bottom electrode. Vectors point outwards, indicating an expansion. Scale bar length, 300 μm. (**d**) Zoom-ins showing the full vector field resolution within the boundary regime (orange box, vectors rescaled by a factor 8) and within a central part in the electrode (blue box, vectors rescaled by a factor 80). Scale bar length, 100 μm. (**e**) Vertical cut through both electrodes and the separator with superimposed vector field (vectors scaled by a factor 8). Scale bar length, 300 μm.

To obtain a qualitative picture of electrode dynamics, we look at cuts C1, C2 and C3 through the electrodes, as indicated by the magenta planes in the sketch in Fig. 2a. Figure 2b shows the horizontal slice C2 through the middle of the top electrode with the superimposed vector field correlating the first with the last scan. Vector arrows point towards an anchor point close to the middle of the electrode, indicating that the electrode contracts within the plane parallel to the current collector (in-plane directions, IP_1_ & IP_2_, Fig. 2a) on delithiation. In contrast, for a slice C3 through the bottom electrode (Fig. 2c), the arrows point outwards, indicating that the electrode expands in the IP directions during lithiation. Supplementary Movie 1 shows the displacement field throughout the whole stack. Figure 2d shows magnifications of the image data and the displacement field in the full computationally available spatial resolution (one vector arrow in 32 × 32 × 32 voxels^3^). The domain taken from the electrode edge (orange box) reveals that the cell housing blocks expansion of the electrode in the IP direction and the domain from the centre of the electrode (blue box) shows a weak random displacement field at the particle scale superimposed on the radial fields describing the overall volumetric changes of the electrode. This indicates that particles and pores of the microstructure slightly deform and reorder with respect to one another during the course of (de)lithiation. The dipole shaped vector field superimposed on the vertical cut C1 through both electrodes and the separator (Fig. 2e) complements the observations from Fig. 2b,c in the TP direction: the glass fibre separator moves upwards, the top electrode contracts, and the bottom electrode expands. The overall volume of the sample remains almost unaffected on a full (de)lithiation, which is expected because of the symmetric cell design and the conservation of charge and mass within the sample.

We further determine that within the graphite electrodes, microstructural changes occur predominantly along the TP direction. The IP vector field components (Fig. 2b,c) are on the order of only 1–2 μm (arrows are scaled by a factor 60), while the TP vector field components (Fig. 2d) represent an average displacement of 10–20 μm (scaling factor 8). The origin of this is twofold: first graphite expands predominantly along its *c*-axis, which corresponds to the shortest dimension of the active particles. As most particles align with their flat side parallel to the current collector (Fig. 1c)^6^, the electrode will favour expansion along the TP direction. Second, from Fig. 2d (vector arrows scaled by a factor of 8), the cell housing constrains further expansion in the IP directions.

To summarize, a qualitative inspection of the vector fields reveals the motion of the electrode and the graphite particles during (de)lithation. In the following sections, we explain how to analyse these vector fields to quantify the electrochemical activity within the electrodes and the corresponding microstructural changes.

### Quantifying electrochemical activity using DVC

Because graphite expands on lithiation, we propose to quantify the electrochemical activity in the electrodes as a function of 3D space and time by calculating the volumetric strain profiles in the electrodes. The relative volumetric change is defined as the divergence of the displacement field, 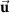
36:


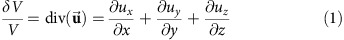


In Supplementary Note 1 and Supplementary Fig. 1 we explain the relations between different types of strain and the displacement field. For example, the dynamic shear strain distribution within the electrodes can be used to detect crack formation at the electrode level.

**Figure 3:**
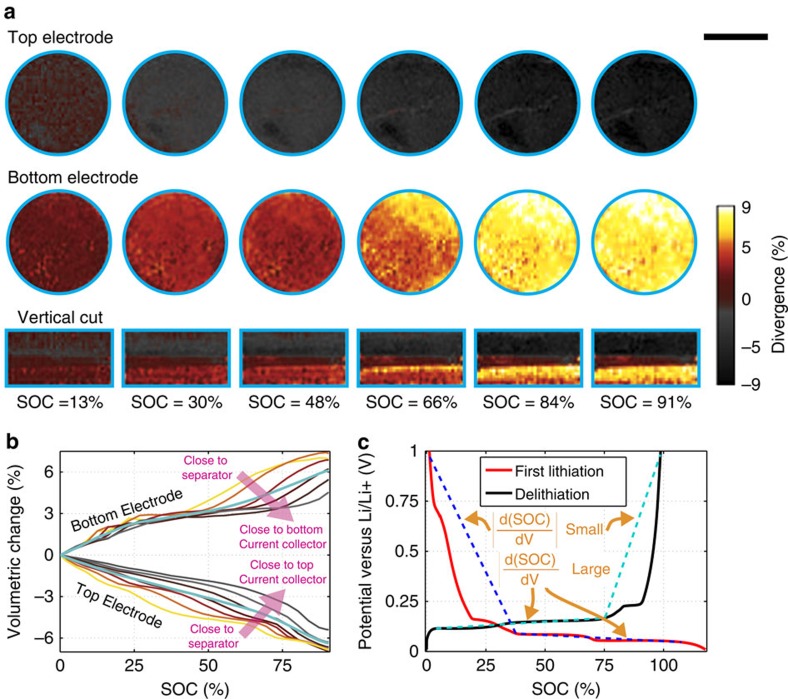
Divergence and strain. (**a**) 3D divergence distribution in the electrodes computed from the displacement field correlating the scans at the indicated times with the first scan (that is, cumulative in time). Scale bar length, 1,000 μm. (**b**) 3D divergence integrated within cylindrical test volumes at various heights in the two electrodes as a function of flowed specific charge. The blue curves represent the average over the whole electrodes. (**c**) First electrochemical cycle of a graphite electrode cycled versus a metallic lithium electrode. The blue dashed lines indicate regimes in the charge- and discharge curves with different slopes.

In Fig. 3a, we map out the volumetric strain distributions within the electrodes as a function of the SOC by correlating the scans at the different SOCs with the 0% SOC scan. The maps indicate that the top electrode (top row) is gradually contracting while the bottom electrode (row in the middle) is expanding. From the vertical cuts (bottom row), it is evident that, up to 48% SOC, volumetric strains occur rather homogeneously in all parts of the lithiating bottom electrode, while at >50% SOC, ‘strain fronts' progress from the electrode-separator interfaces towards the bottom current collector. On the contrary, the delithiating top electrode shows inhomogeneous contraction behaviour, where multiple ‘strain fronts' subsequently progress from the separator–electrode interface towards the top current collector. Supplementary Movies 2–4 show the volumetric strain in all 29 time steps.

To further quantify these observations, we slice the electrodes along planes at equidistantly spaced positions in the TP direction and spatially integrate the divergences of the displacement fields within each of the resulting cylindrical sub-volumes for all time steps. This provides the relative volumetric changes of the electrode sub-sections as a function of the flowed specific charge:


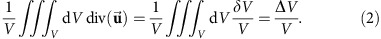


The resulting plot in Fig. 3b shows the per cent volumetric change versus the SOC for the electrode sub-sections at varying distances from the separator. In agreement with our findings from Fig. 3a, we find that both electrodes expand (contract) by ∼6% on average (blue curves) during the course of lithiation (delithiation). Furthermore, we find that (i) the delithiating top electrode contracts progressively along the TP direction, starting from the section closest to the separator and then towards the current collector, and (ii) the lithiating bottom electrode shows a homogeneous expansion throughout the entire electrode up to about 50% SOC followed by a progressive expansion of portions of the electrode beginning with the section closest to the separator.

We now show how DVC-based analysis of volumetric strain can provide insights into electrochemical behaviour. Our analysis leads to two observations: (i) lithiation starts at the electrode–separator interfaces and progresses towards the current collectors and (ii) SOC gradients build-up immediately in the delithiating electrode, while they develop only during the second half of the experiment in the lithiating electrode. The first observation is consistent with our understanding from literature that lithiation kinetics within graphite electrodes are limited by ionic diffusion and conductivity rather than by electric conductivity^11^,^26^,^37^. The second observation can be understood by plotting the first electrochemical cycle of a lithium metal/graphite half cell that has been slowly operated at a galvanostatic C/10 rate (Fig. 3c). During our *operando* experiment, the top electrode will behave similarly to the delithiation curve (black line), while the bottom electrode will behave as the lithiation curve (red line). The larger average slope of the lithiation curve implies that overpotentials developing along the TP direction will induce only smaller SOC gradients within the lithiating bottom electrode, while the smaller slope of the delithiation curve suggests that overpotentials can trigger larger SOC variations within the delithiating top electrode. From the galvanostatic intermittent titration technique protocol (Fig. 1d, inset) used during the experiment, we estimate the overpotential to be approximately 20 mV, or equivalently ΔΦ=10 mV per electrode. According to the calculated slopes, this can lead to SOC variations of 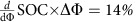 within the top electrode during the first half of the experiment, while the expected SOC variation within the bottom electrode is below 1 %. Only in the second half of the experiment corresponding to the flat regime of the lithiation curve, can gradients also develop in the bottom electrode. The agreement between the expected behaviour of graphite during (de)lithiation and our observations in tomography indicate that for low attenuation contrast and small volume expansion materials such as graphite, DVC is a robust method for tracking small changes in microstructure that can be correlated to electrochemical activity.

### Microstructural analysis on dynamic volumes

Next we demonstrate that our imaging and analysis approach can be used to determine whether the mechanical deformation of the porous electrodes during the (de)lithiation process impacts the transport of lithium ions in the electrode. Here, we focus our analysis on the lithiating bottom electrode.

Suppression of lithium ion diffusion due to microstructure is summarized by:


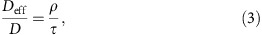


where *D* is the diffusion coefficient of lithium ions in pure electrolyte, *D*_eff_ is the effective diffusion coefficient^3^,^6^, *ρ* is the porosity, and *τ* is the tortuosity^15^,^38^. In contrast to DVC, which operates directly on the raw data, quantification of microstructural parameters, such as porosity or tortuosity, requires data binarization. Additionally, for a dynamically evolving microstructure, determination of microstructural parameters requires the analysis of sub-volumes containing the same particles and pores for each time step.

**Figure 4:**
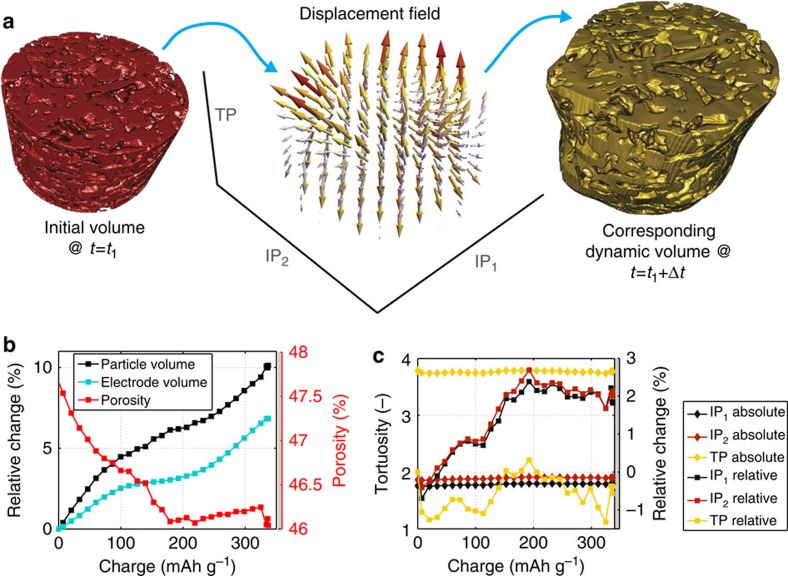
Microstructure analysis on dynamic volumes. (**a**) Left: sketch of a cylindrically-shaped observation volume within the bottom electrode in the initial time step. Middle: vector field representing the displacements within this volume between the first and the last time steps. The IP and TP vector field components have been rescaled for better visibility. Right: dynamic volume in the last time step resulting from warping the initial red volume with the displacement field. (**b**) Electrode volume, particle volume and porosity as a function of charge as determined from the dynamic volumes. (**c**) Tortuosities along the two IP and the TP direction as a function of specific charge. The right axis refers to their change relative to the initial value.

To create dynamic sub-volumes, we use the information readily available from DVC to warp an initial volumetric observation window according to the corresponding computed displacement fields in each time step. This concept is illustrated in Fig. 4a using a small part of the bottom electrode, shown before lithation on the left. During lithiation, the particles in this sub-volume expand, distorting the sub-volume shape. The sub-volume containing the same set of particles is shown in the very last time step (right). For better visibility, the computed displacement field (middle) and the warping effect (right) have been rescaled by a factor 40 in the IP directions and by a factor 6 in the TP direction. Supplementary Movie 5 visualizes this dynamically deforming subvolume.

The chosen *δ*/*β* ratio in the PPC tomography reconstruction provides the appropriate trade-off between resolution and contrast such that each data set can be binarized into a particle phase and an electrolyte/binder/carbon black background phase using Huang's method^39^ (Supplementary Fig. 2 and Supplementary Note 2). In each time step, the threshold that partitions the volumetric grey scale image is calculated by minimizing the Shannon entropy function. As described, a different thresholding method yields similar results, indicating that the binarization is robust (Supplementary Fig. 3 and Supplementary Note 2).

In Fig. 4b, we plot the relative volumetric changes of the dynamic observation window and of the active graphite particles as a function of the flowed specific charge. The overall electrode expands by almost 7% (blue curve), consistent with our findings in Fig. 3b, and the particles (black curve in Fig. 4b) expand by more than 10%, in excellent agreement with literature where it is reported that the crystal lattice of graphite expands predominantly along its *c*-axis direction by approximately 10.5% on a full lithiation^1^,^18^,^19^. The porosity (red curve) first slightly decreases from 48 to 46%, but then remains at 46% for the second half of the lithiation because the particles expand more than the overall electrode only during the first half of lithiation.

Figure 4c shows the absolute and relative values of the tortuosity along the two IP and the TP direction as a function of time. These values have been calculated on a smaller dynamic volume (initial size: 350 × 350 × 250 voxels^3^=227.5 × 227.5 × 162.5 μm^3^), which is sufficiently large for variations due to spatial inhomogeneities in the microstructure to not influence the result (Supplementary Fig. 4 and Supplementary Note 3). The large anisotropy (factor of two) between the IP tortuosities and the TP tortuosity arises from the fact that the graphite particles are platelet shaped and tend to align with their flat-side parallel to the current collector, which results in significantly longer diffusion paths along the TP direction^6^. However, while the TP tortuosity is constant over time, the IP tortuosities increase by ∼2% during the course of lithiation. This effect could arise from the graphite platelets expanding predominately along their c-axis direction, thereby narrowing the diffusion paths along the IP directions. Again, as the rate of electrode expansion overtakes the rate of particle expansion (above 50% SOC), the IP tortuosities no longer increase.

As described in Supplementary Fig. 5 and Supplementary Note 4, use of dynamical volumes enables further types of microstructural analysis techniques.

In summary, for the relatively porous electrode in this cell (designed to keep pressure in order to maintain good electrochemistry, but still accomodating volumetric changes of the sample in the TP direction) lithium transport will not be significantly affected by the reduction in porosity and the narrowing of the pore space (increasing *τ*). The temporal changes in microstructure that occur during lithiation are on the same order of magnitude as the spatial microstructure inhomogeneities across the electrode (Supplementary Fig. 4). However, we emphasize that these finding are electrode and cell specific. Different electrode preparation (for example, lower porosity) or cell configurations (for example, wound versus stacked) could result in sections of the electrode that experience significant changes in the microstructure.

## Discussion

For the case of the graphite electrode, our findings confirm much of what was known or what could be logically hypothesized about (de)lithation of graphite electrodes. We observe that, even in our relatively porous electrodes, lithium transport through the pore space is limited ionically rather than electrically, resulting in a lithation front and large SOC gradients. Expansion in the electrode is strongly anisotropic, stemming from the directional expansion of the graphite flakes that are mostly aligned with their flat side parallel to the current collector. The expansion of the particle phase during lithiation by ∼10% is mostly accommodated by the expansion of the electrode as a whole (6–7%), while the fraction of the active phase increases only slightly, leading to small changes in porosity and tortuosity. These changes in microstructure are on the order of the local inhomogenieties of the graphite electrode itself, and therefore do not significantly worsen the electrochemical performance of the cell. Finally, we observed that the cell housing locally affects the expansion of the electrodes indicating that, depending on the type of cell (pouch or wound), its size, and the packaging materials, the extent of particle expansion that can be accommodated by expansion of the electrode may vary.

Having validated our approach with graphite electrodes, we apply it to study electrodes containing graphite blended with small amounts of silicon (SiC electrodes). SiC electrodes have recently found widespread attention as a solution that enhances the specific charge capacity of the electrode (silicon exhibits a one order of magnitude higher specific charge capacity (3578, mAh g^−1^ (ref. 16)) than graphite (372 mAh g^−1^)), while limiting the volumetric changes of the electrode on electrochemical operation^40^,^41^ (silicon expands by more than 280% (ref. 16) on alloying with lithium, which, when it is the only active material in an electrode, causes severe degradation of the battery performance on prolonged cycling^42^). In fact, only 20 wt% silicon in the electrode are in theory sufficient to triple the specific charge capacity from 372 to 1013, mAh g^−1^. SiC electrodes present similar challenges as graphite electrodes for dynamic imaging in 3D, but, unlike for graphite electrodes, the distribution of electrochemical activity and microstructural dynamics in SiC electrodes are not well understood. Due to the large volumetric change of silicon on (de)lithiation, mechanical deformation within SiC electrodes is expected to be (i) overall significantly stronger and (ii) more heterogeneous compared with pure graphite electrodes.

**Figure 5:**
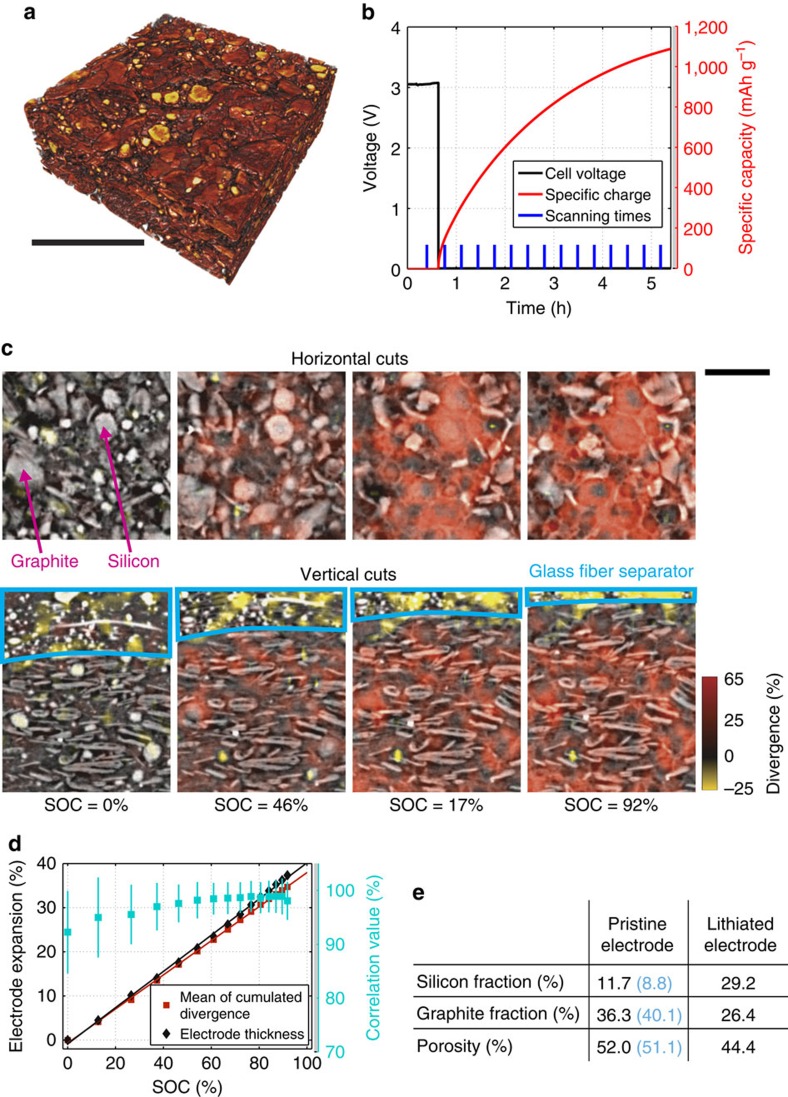
Silicon graphite composite electrode. (**a**) Rendering from a part of the SiC electrode. Silicon particles are shown in yellow and graphite particles are shown in red. (**b**) Specific charge, voltage, and scanning times during *operando* electrochemical operation. The electrode is lithiated at a constant potential of 10 mV. Scale bar length: 100 μm. (**c**) Horizontal cuts (parallel to current collector) and vertical cuts (orthogonal to current collector) through the SiC electrode at different SOCs. The cumulated divergence is shown on top of each image, indicating local expansions (reddish) and contractions (yellowish) according to the scale bar. Scale bar length: 50 μm. (**d**) SiC electrode expansion based on (i) the mean cumulated divergence or alternatively (ii) the electrode thickness along the TP direction as a function of the SOC. The spatial averages of the correlation values at each SOC (blue squares) are an indicator for the goodness of the computed displacement fields. The vertical blue bars display the corresponding standard deviation of the correlation values. (**e**) Silicon, graphite and pore volume fractions in the pristine and the lithiated electrode. The values depicted in black are calculated based on the chemical composition of the electrode while the blue values are obtained from a segmentation of the tomographic data in the three phases.

To investigate the mechanical deformation and electrochemical activity occurring in a SiC electrode during lithiation, as well as the influence of the two types of particle expansion on the microstructure and subsequent electrode performance, we repeat the experiment described above on an electrode containing the same graphite particles mixed with micron-sized silicon particles in a weight ratio of 3:1. Figure 5a shows a rendering of a part of the electrode in the pristine state, where the more attenuating silicon particles are depicted in yellow and the less attenuating graphite particles are depicted in red.

To lithiate the high capacity SiC electrode without the risk of plating on a similar time scale as the graphite electrode, we apply 10 mV potentiostatic conditions right from the beginning until the current has dropped to an effective C/20 rate and the flowed electric charge has reached 93% of the theoretical electrode capacity. Figure 5b summarizes the flowed electric charge and the voltage as a function of time. The blue bars indicate the tomographic scans, which are acquired approximately every 20 min.

To visualize the time- and space-dependent electrochemical activity of silicon and graphite within the electrode, we again turn to DVC and compute the divergence of the displacement fields in each time step. Because electrode deformations and changes in local X-ray attenuation are much larger in the presence of silicon, a smaller SOC increment improves the accuracy of the DVC results. We therefore compute the cumulated divergence at scan number k from the DVC fields 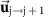 relating subsequent tomographic scans j and j+1, that is:


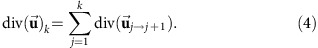


Figure 5c shows the results superimposed on IP and TP cuts through the electrode. We observe a pronounced and clear correlation between material type and expansion characteristic: the domains of strongest expansion (up to 65%) coincide with the positions of the silicon particles. The graphite particles, pore space, and separator expand less or are even compressed by the expanding silicon domains.

Comparing the magnitudes of the displacement fields along the different directions, we find that the electrode expands ∼25 times more in the TP direction as compared with the IP directions. As the expansion mostly stems from spherical polycrystalline silicon particles with isotropic lithiation properties, this anisotropy can only be explained by the cell housing confinement, which limits expansion along the IP directions.

Supplementary Movies 6–8 show horizontal and vertical cuts through the dynamically lithiating SiC electrode superimposed with the corresponding strain distributions.

Figure 5d quantifies the expansion of the SiC electrode using (i) the 3D spatial average of the cumulated divergence within the electrode and (ii) the electrode thickness as determined directly from the tomographic scans (Supplementary Fig. 6 and Supplementary Note 5). The mean correlation values of all DVC computations exceed 90% and the results from the two methods are in excellent agreement with each other. The results indicate that the SiC electrode expansion occurs linearly as a function of the SOC. This is in contrast to graphite, where both the electrode volume and particle volume are nonlinear functions of the SOC (compare ref. 19 and Fig. 4b). Linear expansion as a function of SOC is characteristic for silicon (Supplementary Fig. 7 and Supplementary Note 6). This observation suggests that the electrodes inherit their expansion characteristics from the expansion properties of the constituent particles, and that the expansion of the SiC electrode is dominated by the expansion of silicon.

Finally, we briefly consider the effect of this particle expansion on the microstructure of the SiC electrode. Based on the known chemical composition of the pristine electrode, its mass, its geometric surface area, and its thickness, we estimate (Supplementary Table 1 and Supplementary Note 7) the volume fractions of graphite, silicon, and pore space (Fig. 5e). We compare these results against those obtained from a trinarization (Supplementary Fig. 8 and Supplementary Note 8) of the electrode microstructure in the same three phases (blue numbers). The lithiation of silicon leads to both an expansion of the overall electrode by 35% and a reduction of the porosity by 8%, indicating that, on lithiation, the silicon spreads into the pore space, changing the microstructure, and most likely influencing the lithium transport within the electrodes.

In summary, we demonstrated that a combination of *operando* X-ray tomographic microscopy, PPC, and DVC enables the analysis of the lithiation dynamics and microstructural evolution occurring in LIB electrodes during cycling, thereby guiding the design of next-generation battery electrodes. We validated this combination of techniques on graphite electrodes, which are perhaps the most challenging of all LIB electrodes for X-ray imaging because of the weak attenuation of graphite. We then applied the same set of techniques to study a silicon-graphite composite electrode during the course of lithiation. Our results highlight that DVC can be applied to spatially resolve deformation and electrochemical activity in various types of electrodes. It is suitable for electrodes with Li(Ni_*x*_,Co_*y*_,Mn_1−*x*−*y*_)O_2_ layered structures, LiNi_x_Co_y_Mn_2−*x*−*y*_O_4_ spinels, and LiFePO_4_ olivine structures that, like graphite, undergo small attenuation contrast and volumetric changes in the range 3–15% on lithiation^1^,^20^,^43^,^44^,^45^. Furthermore, our results show that DVC can be used to obtain the dynamic volumes needed for realistic modelling and simulation of 3D electrode microstructures, as well as accurate calculation of microstructural parameters such as porosity and tortuosity as a function of electrode SOC. Quantifying the microstructural changes occurring in an electrode during (de)lithiation enabled us to gain insight into how lithium transport is influenced by the dynamic microstructure. As our results for a SiC composite electrode indicate, this will be a key factor influencing the performance and longevity of next-generation lithium ion battery composite anodes consisting of both, intercalation and alloying compounds.

## Methods

### Experimental cell

An electrochemical cell for *operando* XTM measurements was designed. The battery can consist of two arbitrary electrodes and a separator placed between two stainless steel current collectors. The upper current collector is connected to a spring loaded electrically conductive probe pin, which accommodates for volumetric changes of the electrodes during operation and guarantees a well-defined pressure in TP direction and good electric contact at all times. This pressure is calculated to be approximately 50 N cm^−2^; a typical value for an experimental electrochemical cell^46^. The cell housing is made from polyether ether ketone, an organic thermoplastic polymer with excellent mechanical and chemical properties.

### Sample preparation

For the pure graphite electrodes, slurries were prepared by mixing 3.6 g commercial graphite platelets (Imerys, TIMREX SLP50), 5.333 g 6 wt% PVDF binder (Kynar Flex HSV900) premixed in N-Methyl-2-pyrrolidone (NMP) and 0.08 g Carbon Black (Imerys, C65) in 2 g NMP in a high shear mixer for 10 min (weight ratio 90/8/2 wt%). The slurries were sonicated for 5 min and put on a roll bar for 2 h to remove the remaining air bubbles. Thick electrodes of 250 μm were fabricated by coating a copper foil with the slurry and drying in a vacuum oven at 120 °C for 8 h.

For the silicon graphite mixed electrodes, a combination of 2.4 g graphite (same kind), 0.8 g amorphous/polycrystalline silicon powder (Alpha Aesar, 1–20 μm), 8 g 5 wt% PVDF binder in NMP (same kind) and 0.4 g Carbon Black (same kind) in 6 g NMP was used (weight ratio 60/20/10/10 wt%, 1174, mAh g^−1^ theoretical specific charge capacity). Preparation conditions were the same except for the electrode thickness, which was adjusted to 140 μm.

Free-standing electrodes with a diameter of 1.5 mm were punched out from the electrode sheets. Some of the pure graphite electrodes were pre-lithiated in a standard coin cell at a C/20 rate and a subsequent potentiostatic step at 10 mV. The *operando* cells were then assembled in an argon filled glove box (O_2_<0.1 p.p.m.):

For the graphite–graphite cell, a 1.5 mm pristine graphite electrode was placed facing the bottom current collector, followed by a 250 μm glass fibre separator immersed in 20 μl standard LP30 electrolyte (BASF, 1 M LiPF_6_ in EC:DMC=1:1 by weight) and a pre-lithiated graphite electrode from the coin cell facing the top current collector.

The silicon/graphite-lithium cell was assembled in exactly the same way, except the top electrode was exchanged for a disk punched out from a metallic lithium foil (Alpha Aesar, lithium foil, 99.9%) acting as lithium reservoir. Thereby we avoid the high capacity loss associated with the irreversible capacity of silicon in the first electrochemical cycle.

### Data acquisition

XTM measurements were recorded at the TOMCAT beamline of the Swiss Light Source.

The graphite–graphite sample was scanned continuously, while lithium ions were moved from the top to the bottom electrode at an effective C/7 rate until the cut-off potential at −1.5 V was reached. A galvanostatic intermittant titration technique^33^ protocol was used to provide 57 s long current pulses followed by 3 s relaxation periods. The galvanostatic step was followed by a 45 min potentiostatic step at −1.5 V.

The sample was placed in the beam, 5 cm away from the scintillator to enhance phase contrast. For each scan, 1,201 projections were acquired at 25 keV±2% beam energy using 600 ms integration time per projection, which resulted in 29 scans with a measurement time of ∼17 min each. Using a 10 × microscope and a sCMOS camera with 2560 × 1200 pixels readout, the entire cell (excluding the cell housing wall) could be fit in the resulting 1664 μm × 780 μm field of view with 0.65 μm × 0.65 μm pixel size (local tomography). To ensure comparability between the different scans without the need for image registration techniques, the sample remained mounted in the sample stage in the very same position at all times.

The acquired projections were corrected with flat and dark images. Subsequently, Paganin phase retrieval^29^ was performed with refractive index parameter combinations of *β*=2.25 × 10^−10^ and *δ*=0, (pure attenuation contrast), *δ*=3 × 10^−9^ (used throughout the publication), *δ*=1 × 10^−8^ and *δ*=7.5 × 10^−7^ (strongest retrieval). The filtered projections were reconstructed with Fourier-based reconstruction algorithms implemented at the TOMCAT beamline^47^. With this set of imaging parameters we reach an effective spatial resolution of ∼1.5–2 μm (Supplementary Fig. 9 and Supplementary Note 9).

The SiC electrode was lithiated under potentiostatic conditions at 10 mV (almost short circuit conditions), until the current had dropped to an effective C/20 rate and the flowed electric charge had reached 93% of the theoretical electrode capacity. All image, phase retrieval and reconstruction parameters were identical, except a 20 × microscope was used, resulting in a smaller 832 μm × 390 μm field of view, but higher resolution (0.325 μm × 0.325 μm pixel size). This way, data quality and effective spatial resolution (∼1–1.5 μm, see Supplementary Fig. 9 and Supplementary Note 9) on the microstructural level could be further enhanced and the rather small silicon particles could be better resolved.

### Digital volume correlation

DVC on the graphite–graphite sample was performed on the full reconstructed data sets with the commercial software DaVis8 (LaVision GmbH, Germany), using the direct correlation algorithm with multiple iterations: 6 passes with 256 voxel window size, 48 voxel peak search radius, 8 × 8 × 8 binning, 50% window overlap; 7 passes with 128 voxel window size, 48 voxel peak search radius, 8 × 8 × 8 binning, 50% window overlap and 8 passes with 64 voxel window size, 16 voxel peak search radius, 4 × 4 × 4 binning, 50% window overlap.

In all cases, we used circular shaped interrogation windows and universal outlier detection (remove threshold 2, insert threshold 3, epsilon=0.1, 3 × 3 × 3 neighbourhood). The resulting vector field was smoothed with a Gaussian filter in a 3 × 3 × 3 neighbourhood. For the sketches in Figs 2b,c,e and 4a, the fields were computed with lower resolution for better visibility.

For the silicon/graphite-lithium sample the DVC spatial resolution was pushed to the limit in order to resolve the highly inhomogeneous expansion characteristics stemming from the combination of graphite with silicon: 3 passes with 208 voxel window size, 48 voxel peak search radius, 8 × 8 × 8 binning, 50% overlap; 4 passes with 80 voxel window size, 20 voxel peak search radius, 4 × 4 × 4 binning, 50% overlap and 5 passes with 32 voxel window size, 10 voxel peak search radius, 2 × 2 × 2 binning, 50% overlap. No Gaussian filter was applied to the computed fields. All other settings remained the same.

### Dynamic volumes

An initial cylindrically shaped observation window with a diameter of 1,800 voxels (1,170 μm) and a height of 250 voxels (162.5 μm) taken from the bottom electrode of the graphite–graphite sample was shifted and deformed according to the computed DVC displacement fields of the different time steps using MATLAB's built-in ‘interp3' function. This ‘dynamic mask' then labelled the part of the electrode microstructure in each time step that was used for microstructural computations.

Due to computational limitations, tortuosity calculations were performed on a smaller 350 × 350 × 250 voxels^3^ (=227.5 × 227.5 162.5 μm^3^) cuboid volume, using fixed boundaries in the IP directions and dynamic boundaries in the TP direction. The TP boundary shifts were obtained from the average displacement in TP direction at the boundaries.

### Tortuosity calculations

The tortuosity *τ* was calculated based on the definition and method in ref. 3. The steady state diffusion equation was solved on the full resolution pore space using an in-house code written in MATLAB. To drive particle diffusion through the microstructure along the dimension of interest, different Dirichlet boundary conditions were applied on the boundary planes of the considered volume that are orthogonal on the respective direction. No flux boundary conditions were implied on the other 4 boundaries and all pore—active material interfaces. The linear system of equations was assembled based on the routines developed in ref. 48 and solved iteratively using MATLAB's pre-implemented ‘bicgstabl' iterative solver and incomplete LU factorization (MATLAB's built-in ‘ilu' algorithm).

### Data binarization and trinarization

For the graphite–graphite sample no pre-processing steps were applied to the data. The histograms were computed on the full dynamic observation windows. We cut away the extreme tails of the histograms, by excluding bins with less than 3% of the counts of the histogram maximum. We then used a self-implemented MATLAB version of Huang's Shannon entropy automatic thresholding method^39^ to binarize the data in every time step.

The workflow for the trinarization of the SiC electrode requires more steps and is thus discussed in Supplementary Fig. 8 and Supplementary Note 8.

### Data availability

The data that support the findings of this study are available from the corresponding author on request.

## Additional information

**How to cite this article:** Pietsch, P. *et al*. Quantifying microstructural dynamics and electrochemical activity of graphite and silicon-graphite lithium ion battery anodes. *Nat. Commun.* **7**:12909 doi: 10.1038/ncomms12909 (2016).

## Supplementary Material

Supplementary Information Supplementary Figures 1-9, Supplementary Table 1, Supplementary Notes 1-9 and Supplementary References

Supplementary Movie 1 Visualization of the full displacement field in the graphite - graphite battery. A video showing the full displacement field throughout the graphite electrodes relating the first and the last time steps is available.

Supplementary Movie 2 Volumetric strain in the graphite - graphite battery. Three videos showing horizontal and vertical cuts through the volumetric strain developing in the graphite - graphite battery are available. Part 1

Supplementary Movie 3 Volumetric strain in the graphite - graphite battery . Three videos showing horizontal and vertical cuts through the volumetric strain developing in the graphite - graphite battery are available. Part 2

Supplementary Movie 4 Volumetric strain in the graphite - graphite battery . Three videos showing horizontal and vertical cuts through the volumetric strain developing in the graphite - graphite battery are available. Part 3

Supplementary Movie 5 Movie of the deforming graphite electrode. A movie showing a part of the deforming bottom graphite electrode upon lithiation is available. Deformation effects are scaled for better visibility.

Supplementary Movie 6 Visualization of strain in the SiC electrode. Three videos showing horizontal and vertical cuts through the dynamically lithiating SiC electrode superimposed with the volumetric strain distributions are available. Part 1

Supplementary Movie 7 Visualization of strain in the SiC electrode. Three videos showing horizontal and vertical cuts through the dynamically lithiating SiC electrode superimposed with the volumetric strain distributions are available. Part 2

Supplementary Movie 8 Visualization of strain in the SiC electrode. Three videos showing horizontal and vertical cuts through the dynamically lithiating SiC electrode superimposed with the volumetric strain distributions are available. Part 3
