# Supplementary material for: Quantifying microstructural dynamics and electrochemical activity of graphite and silicon-graphite lithium ion battery anodes
Source: Nat Commun. 2016 Sep 27;7:12909. doi: 10.1038/ncomms12909 (PMC5052642; doi:10.1038/ncomms12909)
Supplement: Supplementary Information — Supplementary Figures 1-9, Supplementary Table 1, Supplementary Notes 1-9 and Supplementary References [file ncomms12909-s1.pdf]

SUPPLEMENTARY FIGURES

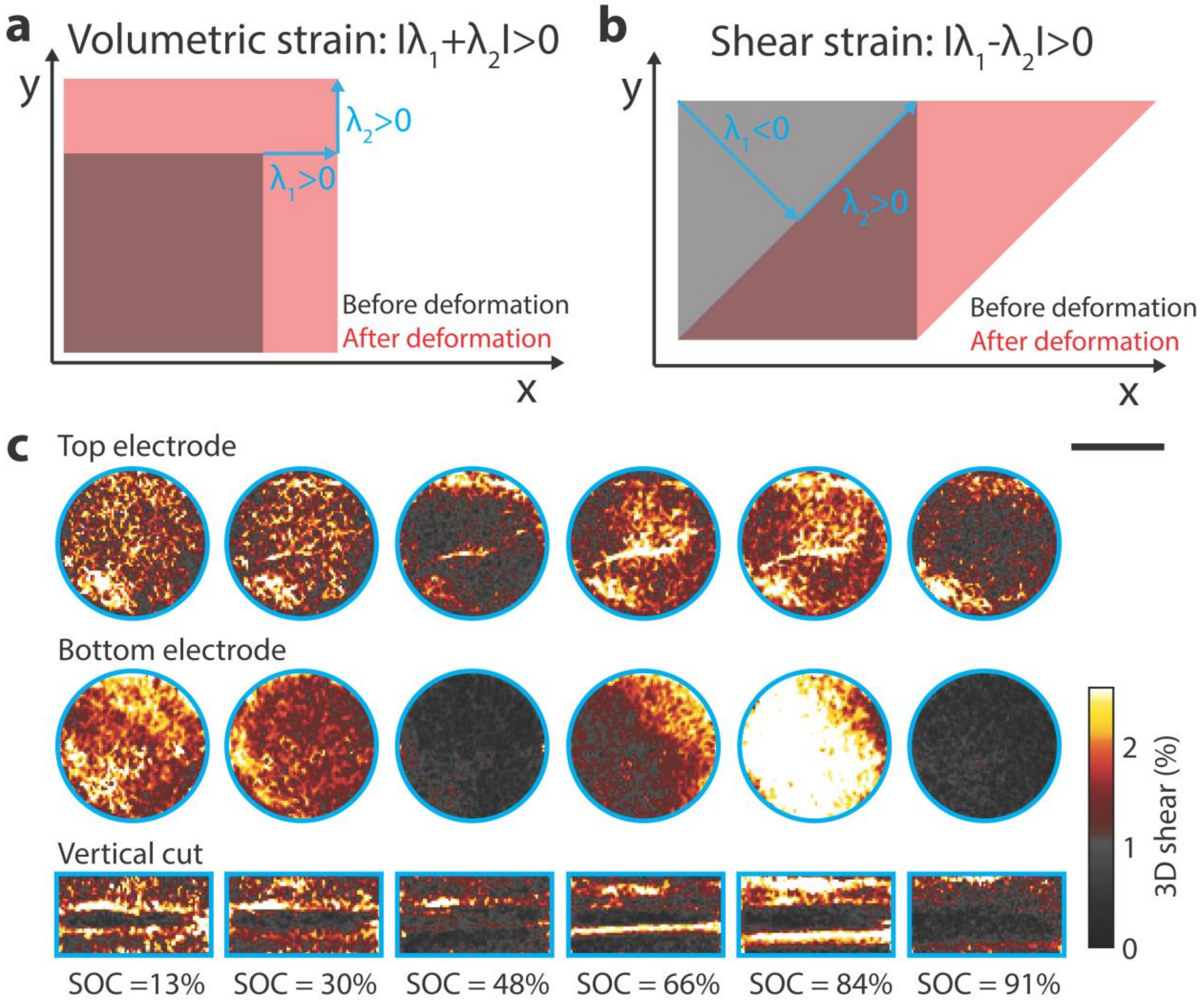

Supplementary Figure 1 | Volumetric and shear strain: (**a**) Sketch illustrating the concept of volumetric strain with λ_1, λ_2 being the eigenvalues of the symmetric strain tensor. The blue arrows indicate the principle directions of the deformation. (**b**) Sketch illustrating the concept of shear strain. (**c**) 3D shear strain distribution in the electrodes computed from the displacement field correlating the scans at the indicated SOCs with the corresponding previous scan shown to the left (i.e. differential in time). Scale bar length: $1000\,\mu m$. See **Supplementary Note 1** for details.

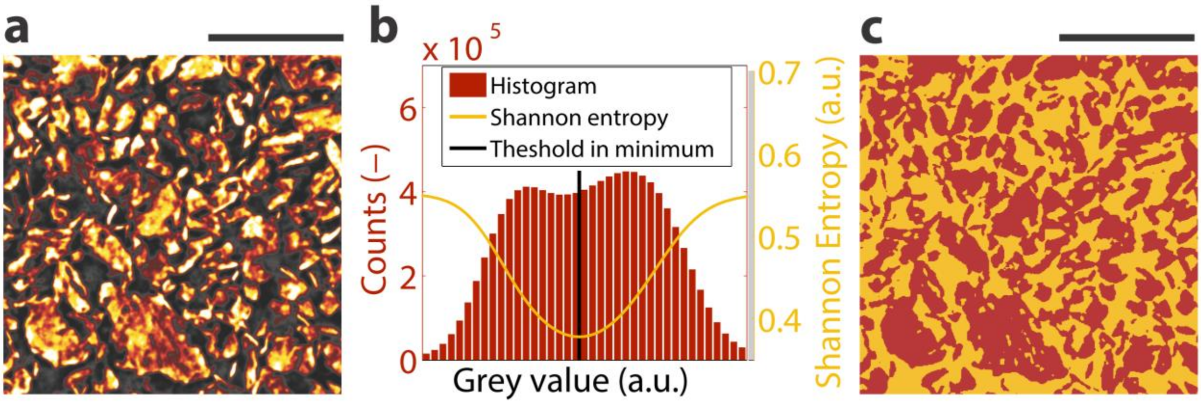

Supplementary Figure 2 | Binarization of the graphite electrode: (**a**) Crop of a slice through the bottom graphite electrode spanned by the IP directions. Scale bar length: $100\ \mu m$. (**b**) Corresponding histogram, Shannon entropy and selected threshold. (**c**) Resulting binarization. Scale bar length: $100\ \mu m$. See **Supplementary Note 2** for details.

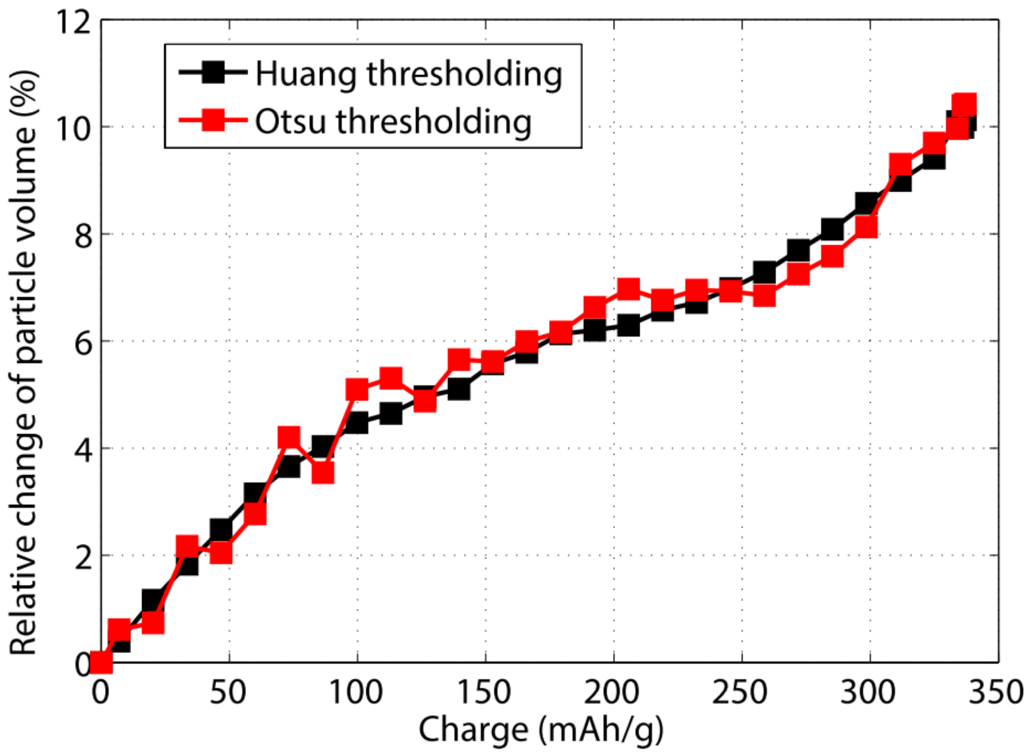

Supplementary Figure 3 | Sensitivity to thresholding algorithm: Relative change in particle volume resulting from binarization using Otsu's and Hunag's thresholding methods. See **Supplementary Note 2** for details.

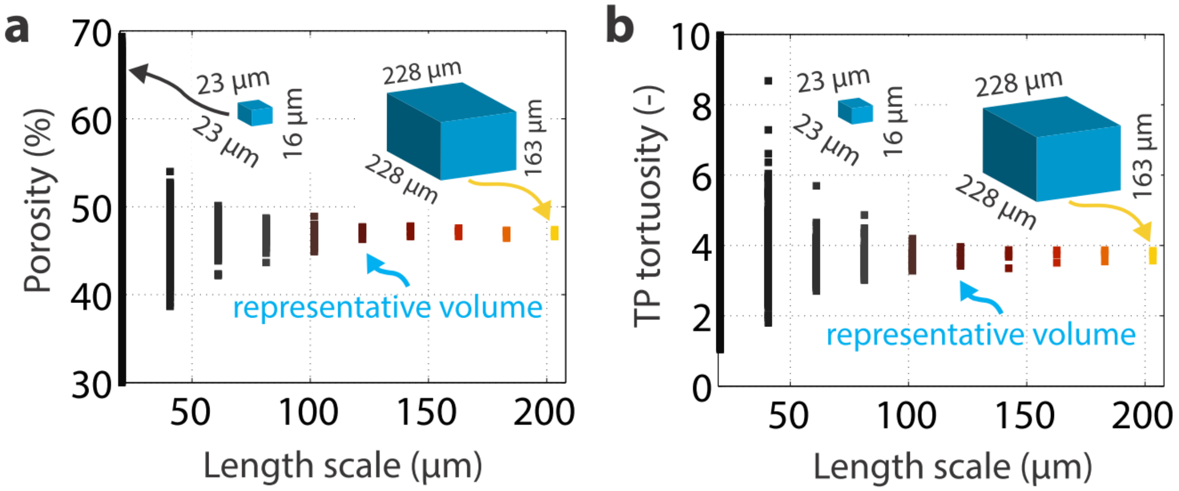

Supplementary Figure 4 | Scale space analysis: (**a**) Porosity ρ calculated on many regularly allocated subvolumes of different sizes at different non-intersecting locations within the bottom electrode microstructure. Each point represents the porosity calculated on one subvolume with a volume equivalent to $(length\ scale)^3$ (**b**) Analog plot for the TP tortuosity τ_{TP}. See **Supplementary Note 3** for details.

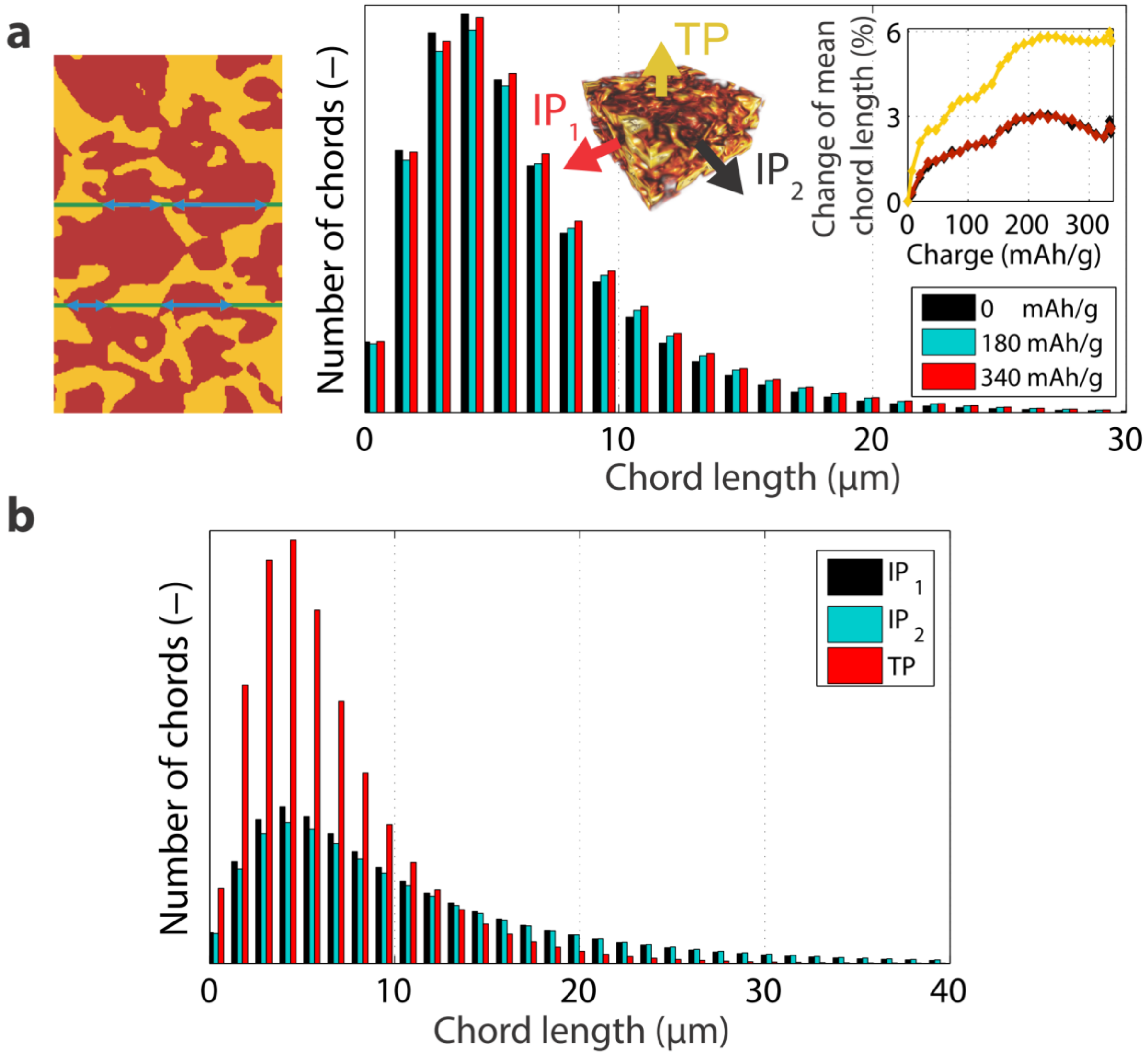

Supplementary Figure 5 | Chord length: (**a**) Left: Sketch illustrating the term "chord lengths". Particles are shown in red, the complimentary phase in yellow. The random lines are shown in green, the blue arrows denote the chords. Right: Distribution of the TP chord lengths for the flowed specific charges as indicated in the legend. Inset: Changes of the mean chord lengths in the two IP and the TP directions during lithiation (see sketch). (**b**) Chord length distributions along the IP and TP directions in the pristine bottom electrode. **See Supplementary Note 4** for details.

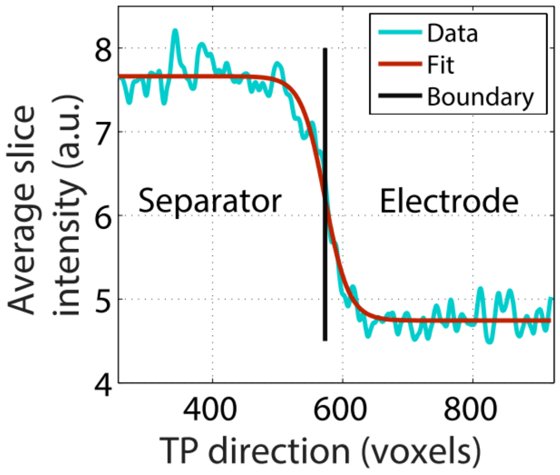

Supplementary Figure 6 | Quantitative determination of the position of the electrode - separator interface: 2D IP spatial grey value average as a function of the TP direction (blue) and corresponding fit (red). The black bar marks the boundary between what is considered electrode and what is considered separator. See **Supplementary Note 5** for details.

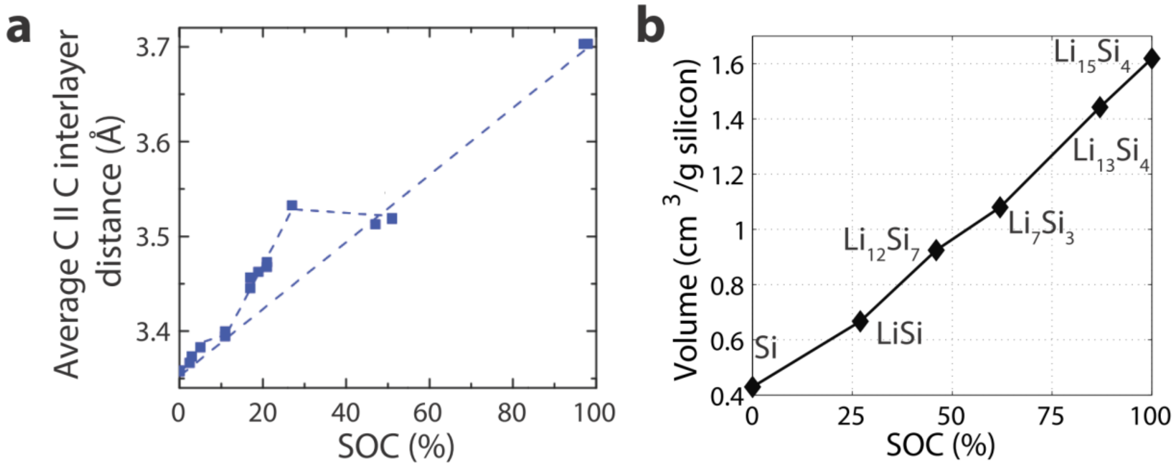

Supplementary Figure 7 | Volume change of silicon and graphite as a function of the state of charge: (**a**) Average C ‖ C interlayer distance in graphite as a function of the SOC measured by neutron diffraction. As expansion within the graphene sheets is negligible, the relative volumetric change of graphite is equivalent to the relative change of the C ‖ C interlayer distance. *Courtesy of Michael Hess*[1]*, page 170 (doi: 10.3929/ethz-a-010000442)*. **(b)** Normalized volume of different Li_xSi_y phases as a function of the SOC. Data points have been calculated based on crystallographic data taken from Chevrier et al.[2]. See **Supplementary Note 6** for details.

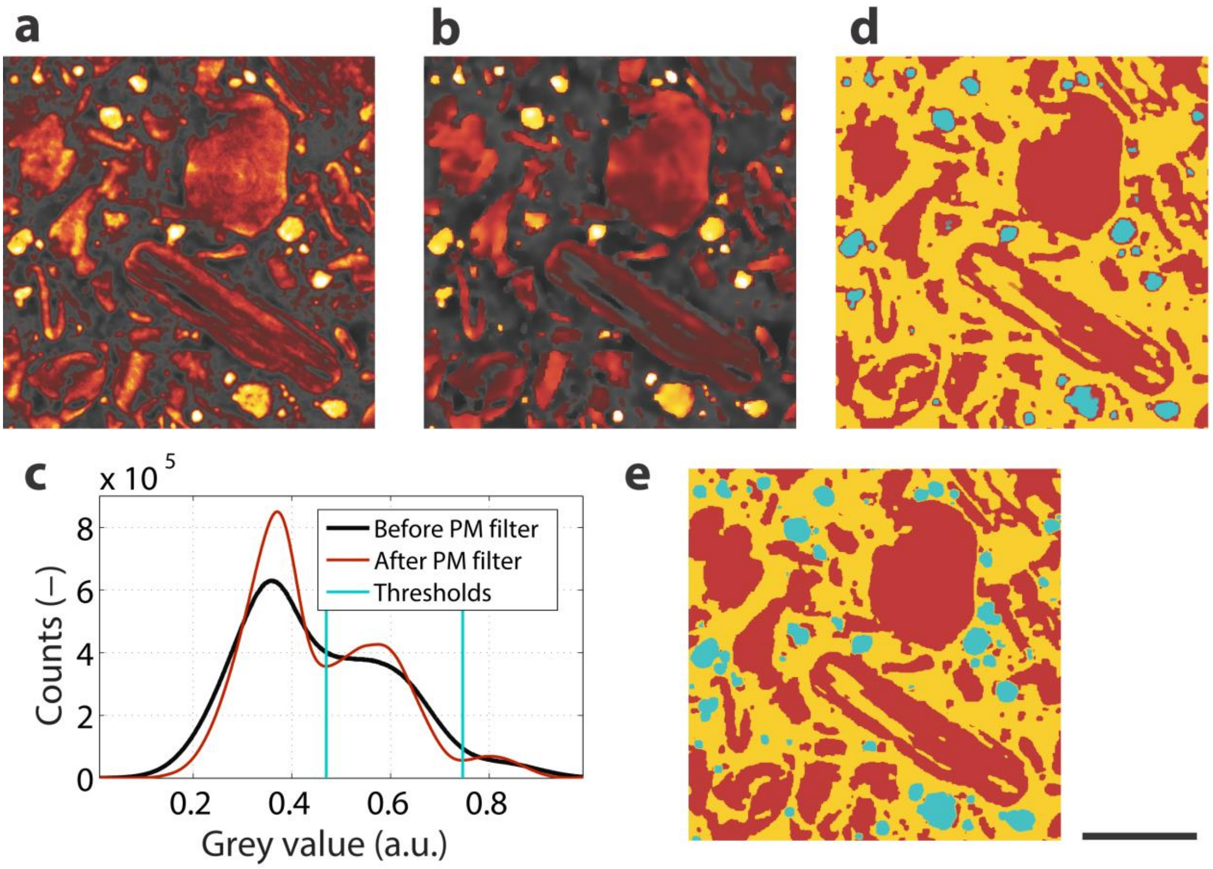

Supplementary Figure 8 | Trinarization procedure for the pristine SiC electrode: (**a**) Original data. (**b**) Data after applying the anisotrop diffusion filter. (**c**) Histograms corresponding to the original (black) and the filtered (red) data. (**d**) Trinarization resulting from thresholding according the values depicted in c). (**e**) Final trinarization after morphological operations. Scale bar length: $50\ \mu m$. See **Supplementary Note 8** for details.

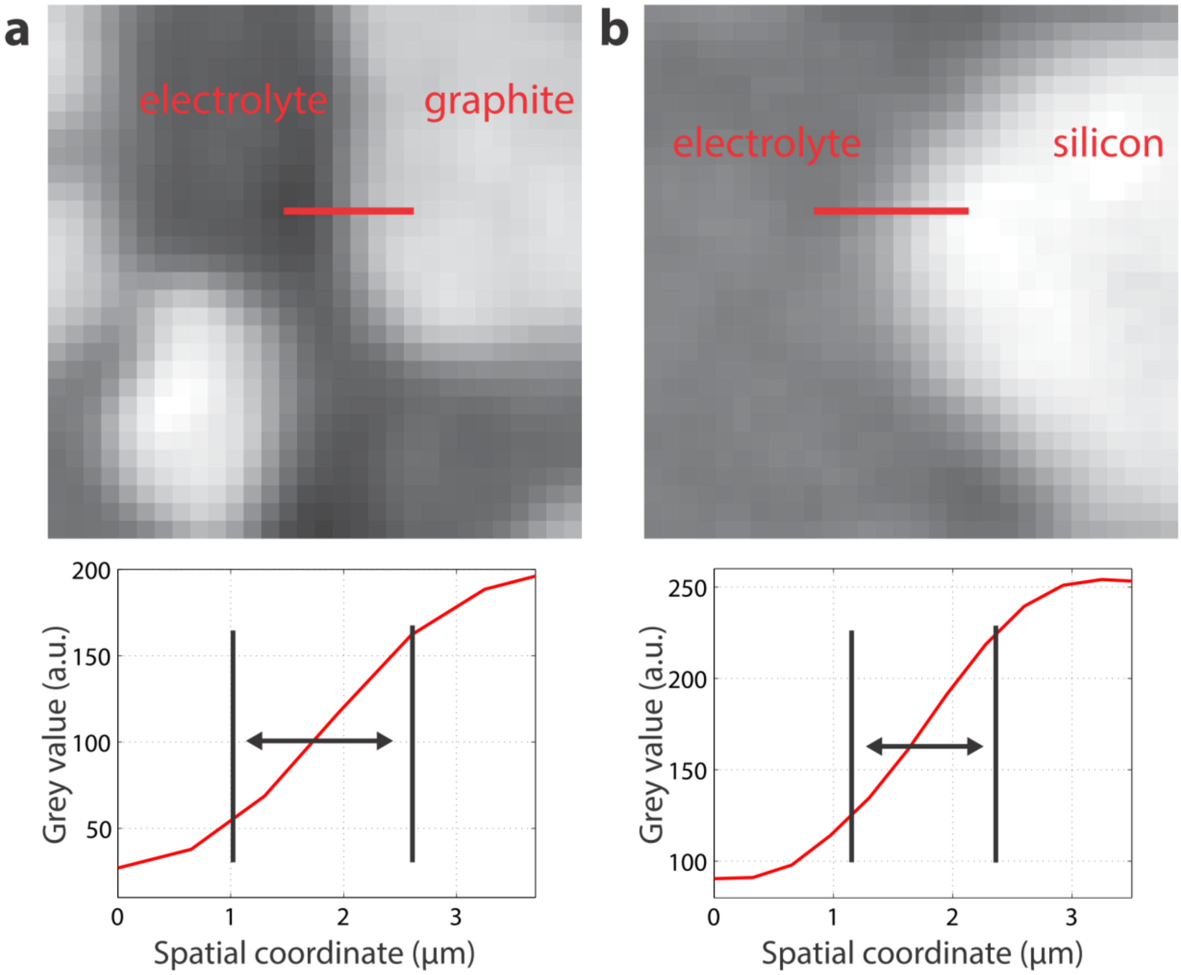

Supplementary Figure 9 | Estimate of spatial resolution: (**a**) Cut through a tomogram recorded with $0.65 \times 0.65 \times 0.65\ \mu m^3$ voxel size. (**b**) Cut through a tomogram recorded with $0.325 \times 0.325 \times 0.325\ \mu m^3$ voxel size. See **Supplementary Note 9** for details.

SUPPLEMENTARY TABLES

Supplementary Table 1. Variables used to describe the calculation of volumetric fractions. See **Supplementary Note 7** for details.

Variable	Value	Meaning
n_{X}, $\mathrm{X} = \mathrm{Si}, \mathrm{Gr}, \mathrm{Pore}, \mathrm{Si(lith.)}, \mathrm{Gr(lith.)}, \mathrm{Pore(lith.)}$	See main text	Volumetric fractions of phase X
f_{Si}, f_{Gr}	20 %, 60 %	Mass fractions (electrode recipe)
ρ_{Si}, ρ_{Gr}	$2.33\ \mathrm{g\ cm}^\wedge - 3$, $2.26\ \mathrm{g\ cm}^\wedge - 3$	Densities of bulk silicon and graphite[3]
γ_{Si}, γ_{Gr}	3.77, 1.105	Volumetric expansion factors for silicon ($\mathrm{Si} \rightarrow \mathrm{Li_{15}Si_4}$)[8] and graphite ($\mathrm{C_6} \rightarrow \mathrm{LiC_6}$)[1,4,5]
m_{El}	240 µg	Electrode mass (measured)
d_{El}, $d_{\mathrm{El(lith.)}}$	99.5 µm, 150.5 µm	Electrode thicknesses at 0 % and 100 % SOC (measured, see **Supplementary Figure 6**)
A_{El}	1.767 mm^2	Geometric surface area of electrode (size of electrode puncher)

SUPPLEMENTARY NOTES

Supplementary Note 1: Volumetric strain and shear strain in the graphite - graphite battery

In infinitesimal strain theory, strain is described by a linearized strain tensor, which can be derived from a given displacement field[6]: $\epsilon_{\mathrm{ij}} = \frac{\partial u_{\mathrm{i}}}{\partial x_{\mathrm{j}}}$. Here, u_{i}, i=1,2,3 are the components of the displacement field and x_{j}, j=1,2,3 are the three space dimensions. ϵ_{ij} may be separated into a symmetric part $\epsilon_{\mathrm{sym,ij}} = \frac{1}{2}\left(\frac{\partial u_{\mathrm{i}}}{\partial x_{\mathrm{j}}} + \frac{\partial u_{\mathrm{j}}}{\partial x_{\mathrm{i}}}\right)$ and an antisymmetric part $\epsilon_{\mathrm{asym,ij}} = \frac{1}{2}\left(\frac{\partial u_{\mathrm{i}}}{\partial x_{\mathrm{j}}} - \frac{\partial u_{\mathrm{j}}}{\partial x_{\mathrm{i}}}\right)$, such that $\epsilon_{\mathrm{ij}} = \epsilon_{\mathrm{sym,ij}} + \epsilon_{\mathrm{asym,ij}}$. While the eigenvalues of the antisymmetric part are imaginary and describe rigid body rotations around the eigenvector directions, the eigenvalues of the symmetric part λ_{i} are real and represent expansions or contractions along the eigendirections. In general, any strain can be expressed as a superposition of a pure volumetric strain component (i.e. the shape of the deformed object remains unchanged) and a pure shear strain component (i.e. the volume of the deformed object remains unchanged).

For better illustration **Supplementary Figure 1** shows the concepts of volumetric strain and shear strain in two dimensions. The eigenvector directions of the linearized symmetric strain tensor are shown as blue arrows in the sketches and point along the principle directions of the deformation. The corresponding eigenvalues λ_{i} indicate whether the object contracts ($\lambda_{\mathrm{i}} < 0$) or expands ($\lambda_{\mathrm{i}} > 0$) along the given direction. A combination of eigenvalues $\lambda_1 = \lambda_2 > 0$ describes a purely volumetric expansion (see **Supplementary Figure 1a**), while a combination $\lambda_1 = \lambda_2 < 0$ describes a purely volumetric contraction. In analogy, a combination $\lambda_1 = -\lambda_2$ describes a pure shear deformation, where the volume of the object remains unchanged (see **Supplementary Figure 1b**). In two dimensions any strain contains a volumetric component, if $|\lambda_1 + \lambda_2| > 0$ and a shear component, if $|\lambda_1 - \lambda_2| > 0$.

More generally, in three dimensions, volumetric strain can be described by the divergence of the displacement field or equivalently the trace of the symmetric strain tensor, which is the sum of its eigenvalues:

$$\frac{\delta V}{V} = \mathrm{div}(\vec{\mathbf{u}}) = \frac{\partial u_{\mathrm{x}}}{\partial x} + \frac{\partial u_{\mathrm{y}}}{\partial y} + \frac{\partial u_{\mathrm{z}}}{\partial z} = \mathrm{Tr}\left[\bar{\bar{\boldsymbol{\epsilon}}}_{\mathbf{sym}}\right] = \sum_{\mathrm{i}} \lambda_{\mathrm{i}} \tag{1}$$

In analogy, the shear strains along the different dimensions correspond to the respective differences of the eigenvalues, i.e.:

$$S_{ij} = \left|\lambda_i - \lambda_j\right|. \tag{2}$$

For our computations, linearized strain theory is applicable because $\left|\epsilon_{\mathrm{ij}}\right| < \frac{1}{20} \ll 1$ holds for the computed displacement fields for any time and position. For more information, we refer the reader to [6].

Three videos showing the volumetric strain in all 29 time steps are available.

To complement the analysis of volumetric strain presented in the main text, we also investigate the distribution of maximal shear in the electrodes, which we define as:

$$S_{max} = max_{i,j}\left(\left|\lambda_i - \lambda_j\right|\right). \quad (3)$$

Shear strain is expected to occur in the electrodes because (i) the expansion of graphite upon intercalation of lithium is anisotropic and (ii) the cell housing limits further expansion only along the IP directions. Because of (i), shear strain will be correlated with electrochemical activity and volumetric strain.

To achieve time resolution, we compute the shear for the displacement field correlating subsequent scans (i.e., the initial 0 % SOC scan with the 13 % SOC, the 13 % SOC scan with the 30 % SOC scan, and so on).

Supplementary Figure 1c depicts this time series of shear strains for slices in the top and bottom electrodes and a vertical cut through both electrodes and the separator respectively. In agreement with the observations from **Figure 3a** in the main text, shear strain in the TP direction progresses through the bottom electrode along with the moving lithiation front, while shear strain is more uniform along the TP direction in the top electrode, indicating that delithiation is occurring more homogeneous here. Moreover, the shear strain is more homogenous along the IP directions of the lithiating bottom electrode, compared to the delithiating top electrode, where a large crack-like feature can be observed. This suggests that the analysis of local strains might enable the identification of sub-domains that become mechanically and electrochemically disconnected from the electrode. This would result in capacity fade.

Supplementary Note 2: Binarization of the bottom graphite electrode

The dynamically cropped volumes are binarized into a particle phase and an electrolyte/binder/carbon black background phase using Huang's automatic thresholding method[7]. In each time step, the threshold that partitions the volumetric grey scale image is calculated by minimizing the Shannon entropy function. **Supplementary Figure 2a** shows a crop of a raw data slice for illustration purposes. The corresponding histogram and the Shannon entropy function are shown in **Supplementary Figure 2b.** Selecting the threshold at the position of the black bar results in the binarization shown in **Supplementary Figure 2c.**

In general, the thresholding method may significantly influence the resulting data binarization and all microstructure analysis that is based upon it. In order to test the sensitivity of our binarization on the type of thresholding algorithm applied, we compare the particle volume change as a function of flowed charge for two different thresholding methods: Huang's (used throughout the main paper) and Otsu's[8]**.**

As evident from **Supplementary Figure 3,** the results differ insignificantly, demonstrating that our analysis is robust.

Supplementary Note 3: Scale space analysis of the graphite-graphite battery

To determine a representative volume on which the microstructural parameters should be calculated, we performed a scale space analysis. **Supplementary Figure 4** shows the porosity ρ (**Supplementary Figure 4a**) and the through-plane tortuosity τ_{TP} (**Supplementary Figure 4b**) as a function of sub-volume size. For this analysis, a $(700 \times 700 \times 250)$ voxel^3 $=$ $(455 \times 455 \times 163)$ μm^3 large volume was cropped out from the binarized microstructure of the pristine bottom electrode. ρ and τ_{TP} were then computed on non-overlapping regularly allocated sub-volumes of sizes a $\times$ $(35 \times 35 \times 25)$ voxels^3 $=$ a $\times$ $(3\ \times 23 \times 16)$ μm^3 with a=1…10 within this volume. Each point in the **Supplementary Figure 4a-b** represents the results obtained from one sub-volume with a size as indicated on the x-axis. As the sub-volume size is increased, the distributions in the microstructure converge towards a porosity $\rho \approx 47$ % and a tortuosity $\tau_{\mathrm{TP}} \approx 3.7$.

As indicated by the blue arrow, subvolumes with an edge length equal or greater than approximately 3 times the long axis of an average particle (i.e. $(185 \times 185 \times 185)$ voxel^3 $=$ $(120 \times 120 \times 120)$ μm^3) are representative of the full electrode microstructure and reveal similar spatial variations in the microstructural parameters. This indicates that there is some small microstructural inhomogeneity at length scales larger than 200 μm.

For the tortuosity calculations in the main text, a volume of $(350 \times 350 \times 250)$ voxel^3 $=$ $(228 \times 228 \times 163)$ μm^3 is considered. We note that the spatial variations in the tortuosities (< 10 %) are on the same order of magnitude as the temporal variations in tortuosities that occur during lithaition. The same holds for the porosities. This underscores the necessity to consider dynamic observation windows.

Supplementary Note 4: Chord length analysis of the bottom graphite electrode

To show that different types of microstructural analysis can be applied to our imaged graphite electrode, we consider another approach that is well-known in statistical image analysis: the so-called chord length distribution[9,10]. The concept is sketched in the left part of **Supplementary Figure 5a**. If one intersects a random line (green) with a given direction (here, either IP or TP direction) with the foreground in the image (red), so-called chords (blue) originate. The distribution of the length of these chords is called chord length distribution. For more detailed information, we refer the reader to [11]. **Supplementary Figure 5a**, right shows a histogram of the resulting chord lengths in TP direction for the first measurement with 0 % SOC, an intermediate one with about 48 % SOC and the last one with about 91 % SOC. Between 0 % SOC and 48 % SOC, particles are growing and a shift towards longer chords can be observed in the histogram (i.e. the number of smaller chords less than about 7 μm decreases with time while the number of chords larger than 7 μm increases). Accordingly, the inset shows how the mean chord length in TP direction is growing by approximately 6 %. This effect is qualitatively similar but less expressed in the IP directions, where the mean chord length increases by only 3% (see inset of **Supplementary Figure 5a**).

Between 48 % SOC and 91 % SOC the chord lengths continue to increase on average, but this effect is counter-balanced by two other effects: (i) the expansion of the particles in the IP directions gives rise to new short chords at the particle boundaries and (ii) the strong expansion of the electrode in TP direction during the second half of the lithiation gives rise to a separation of chords that have been previously connected. For this reason, the mean chord length stagnates in this regime.

Finally, **Supplementary Figure 5b** shows the chord length distributions along the different dimensions in the pristine electrode. Chords along the TP direction are on average much shorter than their IP counterparts, demonstrating that the chord length distribution is an excellent tool to characterize the anisotropic shape of our graphite particles.

Supplementary Note 5: Volume expansion of the SiC electrode based on its thickness

If the electrode-current collector interface is assumed to remain at the same position at all times and expansions along the IP directions are neglected, the relative volumetric change of the electrode can be described as its relative thickness change along the TP direction (i.e. the average movement of the electrode – separator interface along the TP direction).

To quantitatively find the position of the electrode - separator interface in each time step, we computed the 2D IP spatial grey value average as a function of the TP direction. Because the separator always appears brighter than the electrode, this results in a step-like function (see blue curve in **Supplementary Figure 6**).

We perform a least-squares fit to the data using a function of type:

$$f(x) = G_2 + \frac{G_1 - G_2}{1 + e^{k \cdot (x - \mu)}}, \qquad (4)$$

where G_1, G_2, k and μ are fit parameters (see red curve in **Supplementary Figure 6**). μ denotes the TP position corresponding to the average of the separator grey value level G_1 and the electrode grey value level G_2 (i.e. $f(\mu) = \frac{G_1 + G_2}{2}$). We consider μ as a measure of the electrode-separator interface position and hence the electrode thickness in each time step (see black bar in **Supplementary Figure 6**).

Supplementary Note 6: Volume change of silicon and graphite as a function of the state of charge

Graphite has been shown to exhibit an “S-shaped” expansion characteristic (**Supplementary Figure 7a**) while silicon expands linearly as a function of the lithium content (**Supplementary Figure 7b**)[12]. We note that the curves in **Figures 4b** and **5d** in the main text showing the volume expansion of the graphite and SiC electrodes, respectively, qualitatively resemble the volume expansion of silicon and graphite as a function of the degree of lithiation. The observed SOC gradients within the electrodes and the particles explain why small features are blurred out in the data presented in **Figures 4b** and **5d**.

Supplementary Note 7: Volumetric fractions of silicon, graphite and pore space in the SiC electrode

The volumetric fractions of (lithiated) silicon, (lithiated) graphite and pore space in the SiC electrode can be estimated based on the mass of the electrode, its chemical composition, its geometric surface area and its thickness. Variables are defined in **Supplementary Table 1**.

For the pristine electrode, we find:

$$n_{Si} = \frac{f_{Si} m_{El}}{\rho_{Si} d_{El} A_{El}} \tag{5}$$

$$n_{Gr} = \frac{f_{Gr} m_{El}}{\rho_{Gr} d_{El} A_{El}} \tag{6}$$

$$n_{Pore} = 1 - n_{Si} - n_{Gr}. \tag{7}$$

For the lithiated electrode, we find:

$$n_{Si(lith.)} = \frac{\gamma_{Si} f_{Si} m_{El}}{\rho_{Si} d_{El(lith.)} A_{El}} \tag{8}$$

$$n_{Gr(lith.)} = \frac{\gamma_{Gr} f_{Gr} m_{El}}{\rho_{Gr} d_{El(lith.)} A_{El}} \tag{9}$$

$$n_{Pore(lith.)} = 1 - n_{Si(lith.)} - n_{Gr(lith.)}. \tag{10}$$

Supplementary Note 8: Trinarization procedure for the pristine SiC electrode

We aim to trinarize the tomographic data of the SiC electrode to identify the three phases: silicon, graphite, and pore phase.

Supplementary Figure 8a shows a cutout from a slice through the electrode along the IP directions. The histogram of gray level values (see **Supplementary Figure 8c** black curve) does not show two clear local minima, indicating that given the noise level, the contrast between the three phases is insufficient to select a set of thresholds.

Therefore, to trinarize the tomographic data, we apply a series of image processing steps using the software MATLAB.

First, we apply an anisotrop diffusion filter[13] (parameters:$g(|\nabla I|) = \exp\left(-\left(\frac{|\nabla I|}{K}\right)^2\right)$, K according to the Canny noise estimator[14]; 40 iterations with time step $dt = 0.2$) that equalizes grey values within smooth domains while preserving sharp edges at interfaces between different phases (see **Supplementary Figure 8b**). The resulting histogram (see **Supplementary Figure 8c** red curve) shows two clear local minima, which we chose as thresholds for a trinarization shown in **Supplementary Figure 8d (**automatic thresholding algorithms like Otsu's[8] or Huang's[7] method fail in this case as the phase fractions are very

unequal). Finally, we correct for the rings around the silicon particles that are inevitable because the silicon-pore interface domains are not infinitely sharp and are thus falsely identified as graphite. We do so by using a set of morphological operations in MATLAB (dilation of the silicon domain using a ball structuring element of radius 3 voxels, closing of the silicon domain using a ball structuring element of radius 2 voxels, dilation of the graphite domain using a ball structuring element of radius 1 voxel) to arrive at the final trinarization shown in **Supplementary Figure 8e**.

The trinarization (see **Figure 5e** in the main text) tends to underestimate the silicon fraction (8.8 % instead of the actual 11.7 %) because small silicon particles are either below the resolution limit or are falsely associated with the graphite domain. However, the trinarization captures the major features of large particles well.

A trinarization of the data for (partially) lithiated states is not possible due to insufficient contrast: as seen in **Figure 5c** in the main text, the X-ray attenuation of fully lithiated silicon particles is comparable to the attenuation of the background.

Supplementary Note 9: Estimate of spatial resolution

Tomographic scans of the graphite-graphite sample have been performed with an effective voxel size of $0.65 \times 0.65 \times 0.65\ \mu m^3$, while scans of the sample with the silicon-graphite electrode were recorded with a voxel size of $0.325 \times 0.325 \times 0.325\ \mu m^3$.

We estimate the effective spatial resolution, i.e. the ability to distinguish two objects that are close to each other from one another, by plotting grey value profiles through supposedly sharp edges in the images (particle – electrolyte interface) and analyzing their width (see **Supplementary Figure *9*a-b**). Depending on the definition of resolution used, we find around $1.5 - 2\ \mu m$ spatial resolution for $0.65\ \mu m$ voxel size and $1 - 1.5\ \mu m$ spatial resolution for the $0.325\ \mu m$ voxel size.

Supplementary References

1. Heβ, M. Kinetics and stage transitions of graphite for lithium-ion batteries. *Ph.D. thesis, ETH Zurich,* 1–209 (2013). doi:10.3929/ethz-a-010000442

2. Chevrier, V. L., Zwanziger, J. W. & Dahn, J. R. First principles study of Li–Si crystalline phases: Charge transfer, electronic structure, and lattice vibrations. *J. Alloys Compd.* **496,** 25–36 (2010).

3. Doh, C.-H., Han, B.-C., Jin, B.-S. & Gu, H.-B. Structures and formation energies of LixC6 (x=1-3) and its homologues for lithium rechargeable batteries. *Bull. Korean Chem. Soc.* **32,** 2045–2050 (2011).

4. Ohzuku, T., Iwakoshi, Y. & Sawai, K. Formation of lithium-graphite intercalation compounds in nonaqueous electrolytes and their application as a negative electrode for a lithium ion (Shuttlecock) cell. *J. Electrochem. Soc.* **140,** 2490–2498 (1993).

5. Dahn, J., Fong, R. & Spoon, M. Suppression of staging in lithium-intercalated carbon by disorder in the host. *Phys. Rev. B.* **42,** 6424–6432 (1990).

6. Lai, W. M., Rubin, D. & Krempl, E. *Introduction to Continuum Mechanics*. (Butterworth-Heinemann, 2010).

7. Huang, L. K. & Wang, M. J. J. Image thresholding by minimizing the measures of fuzziness. *Pattern Recognit.* **28,** 41–51 (1995).

8. Otsu, N. A threshold selection method from gray-level histograms. *IEEE Trans. Syst. Man. Cybern.* **9,** 62–66 (1979).

9. Westhoff, D. *et al.* Stochastic modeling and predictive simulations for the microstructure of organic semiconductor films processed with different spin coating velocities. *Model. Simul. Mater. Sci. Eng.* **23,** 045003 (2015).

10. Feinauer, J. *et al.* Stochastic 3D modeling of the microstructure of lithium-ion battery anodes via Gaussian random fields on the sphere. *Comput. Mater. Sci.* **109,** 137–146 (2015).

11. Chiu, S. N., Stoyan, D., Kendall, W. S. & Mecke, J. *Stochastic Geometry and its Applications*. (J. Wiley & Sons, Ltd, 2013).

12. Pietsch, P., Hess, M., Ludwig, W., Eller, J. & Wood, V. Combining operando synchrotron X-ray tomographic microscopy and scanning X-ray diffraction to study lithium ion batteries. *Sci. Rep.* **6,** 27994 (2016).

13. Perona, P. & Malik, J. Scale-space and edge detection using anisotropic diffusion. *IEEE Trans. Pattern Anal. Mach. Intell* **12,** 629–639 (1990).

14. Canny, J. A Computational Approach to Edge Detection. *IEEE Trans. Pattern Anal. Mach. Intell.* **8,** 679–698 (1986).
